# Increasing Drunkeness in England

**Published:** 1871-05

**Authors:** 


INCREASING DRUNKENNESS IN ENGLAND.—In the Manchester district, the number of commitments to jail for drunkenness, in proportion to other offences, was: in 1866, 26¾ per cent.; in 1867, 27¼ per cent.; in 1868, 29 per cent.; in 1869, 30 per cent.; in 1870, 37 per cent. It might be well for medical men, who are in the habit of prescribing and encouraging the use of intoxicating drinks, to reflect whether there is any relation between this growth of intemperance and the increased use of alcohol for medicinal and dietetic purposes under professional sanction. Many of the most eminent British physicians have spent much labor, in the last twenty years, to expose the errors and follies of the teetotalers, and to prove that total abstinence is unfavorable to health. We venture to propound two inquiries, which we deem pertinent to the subject, and which may be answered by *guessing:* First, in view of the foregoing figures, how many persons in the Manchester district have suffered in health and life, during the last year, by the use of strong drink? Second, how many persons in the same district have suffered in health and life by drinking only water? Neither of these questions admits of a definite answer, but the endeavor to answer them suggests a third: If the claim of our profession to philanthropy be well founded, would it not be well to reserve a few tears for the victims of intemperance, and not exhaust the supply over a few individuals who are supposed to endanger their health by drinking water?—*Pacific Medical Journal.*

